# A novel handheld wireless robot for total knee arthroplasty: Early experiences and clinical results

**DOI:** 10.1002/jeo2.70444

**Published:** 2025-09-29

**Authors:** Alexander P. Sah, James L. Womack, Yair D. Kissin, Eytan M. Debbi, Trevor R. Cotter

**Affiliations:** ^1^ Washington Hospital Healthcare System Fremont California USA; ^2^ Golden Valley Memorial Healthcare Clinton Missouri USA; ^3^ Hackensack University Medical Center Hackensack New Jersey USA; ^4^ Hospital for Special Surgery New York New York USA; ^5^ THINK Surgical, Inc. Fremont California USA

**Keywords:** clinical outcomes, knee, orthopaedics, robotic surgery, total knee arthroplasty

## Abstract

**Purpose:**

The percentage of total knee arthroplasty (TKA) performed robotically (rTKA) is rising exponentially. While this technique has well‐established accuracy improvements, there are mixed patient‐reported outcome measure (PROM) benefits compared to manual TKA (mTKA). Potential challenges in workflow, restricted implant compatibility to the robot and system cost have limited robotic adoption. A new robot seeks to address these and other rTKA concerns.

**Methods:**

This was a single surgeon retrospective series of the first 25 rTKA cases performed with a novel, wireless, handheld, open‐implant robot and 25 mTKA cases. Data were recorded to observe performance and safety outcomes, including X‐ray‐based hip‐knee‐ankle (HKA) angle measurements, adverse events (AEs), range of motion (ROM), and PROMs including pain and knee society score (KSS). Case times for a subset of cases were recorded. rTKA cases were completed with implants not previously used by the surgeon. mTKA cases used implants and instrumentation standard to the surgeon's practice. Patients were tracked up to 3 months.

**Results:**

rTKA and mTKA cases both yielded positive clinical results. There were 0/25 HKA angle outliers (>3° from plan) and 1/25 outliers in the rTKA and mTKA cohorts, respectively. No AEs were observed in either group at follow‐up. rTKA case times were similar to mTKA by case 5, with rTKA setup under 8 min for most cases. There were no differences in ROM or PROMs between cohorts.

**Conclusion:**

rTKA preliminarily showed safe and effective outcomes in early results. Surgeon and staff adoption showed minimal hurdles at this institution, as rTKA case with an implant system new to the surgeon were similar within five cases to mTKA case times using an implant system known to the surgeon, with comparable clinical results. This initial experience suggests the system may offer a more accessible alternative to existing robotic options, though further study is needed.

**Level of Evidence:**

Level IV.

**Abbreviations**
CT: computed tomography scans
KSS: knee society score
mTKA: manual total knee arthroplasty
OR: operating room
PROM: patient‐reported outcome measures
ROM: range of motion
rTKA: robotic total knee arthroplasty
TKA: total knee arthroplasty
VAS: visual analog scale

## INTRODUCTION

Total knee arthroplasty (TKA) has become a procedure that is commonly performed robotically (rTKA), with use of robotics expected to increase [10]. Surgeons may choose robotics for TKA for a variety of reasons, including the potential for increased accuracy compared to manual procedures (mTKA) [18, 25]. rTKA may result in better knee alignment and range of motion (ROM) for patients [19, 25]. Many rTKA systems currently on the market allow for preplanning of cases using preoperative computed tomography (CT) scans to assist surgeon preparation for the procedure. This additional efficiency may help hospitals by lowering inventory costs and intraoperative modifications [3].

Despite the potential advantages of rTKA, over 80% of TKAs performed in the United States each year are still performed manually with traditional instruments [14]. There may be several reasons for this. First, this may be because conclusive clinical data proving better patient outcomes for rTKA is still lacking. Studies comparing patient‐reported outcome measures (PROMs), such as the knee society score (KSS), between mTKA and rTKA have shown minimal or no statistically significant differences between the two techniques [2]. Additionally, most rTKA systems are closed‐implant, meaning that only the knee implants of the company selling the robot can be used with the robot. This limits surgeon choice by generally preventing surgeons from using an implant they think best for their patient and instead having to opt for an implant made by the robot manufacturer.

Another limitation to adoption may be that robotics can be disruptive to workflow in the operating room (OR). rTKA generally has a workflow that differs from a typical mTKA workflow, which forces hospital staff and surgeons to learn new techniques and tools, such as robotic calibration, saw attachment, and more, and may contribute to increased cognitive load and workflow disruption in the OR [20]. Robotic procedures tend to be more cumbersome, due to sterility concerns from cables and attachments [8, 26], large footprints [8, 28, 37] and sensitivities to line of sight obstructions that prevent staff from moving normally throughout the OR and sterile field [8, 26, 28, 37]. These drawbacks may lead to increased operating time, which has been linked to infection risk and increased operating costs [16, 20]. While surgeons and staff are trained to ensure the first patients treated with a given procedure or technology receive appropriate care, these early cases still carry increased risks and stress [17, 30]. However, despite these risks, robotics are common today and expected to be used in half of all knee arthroplasties by 2030, therefore systems should seek to become less disruptive in the OR [10].

**Figure 1:**
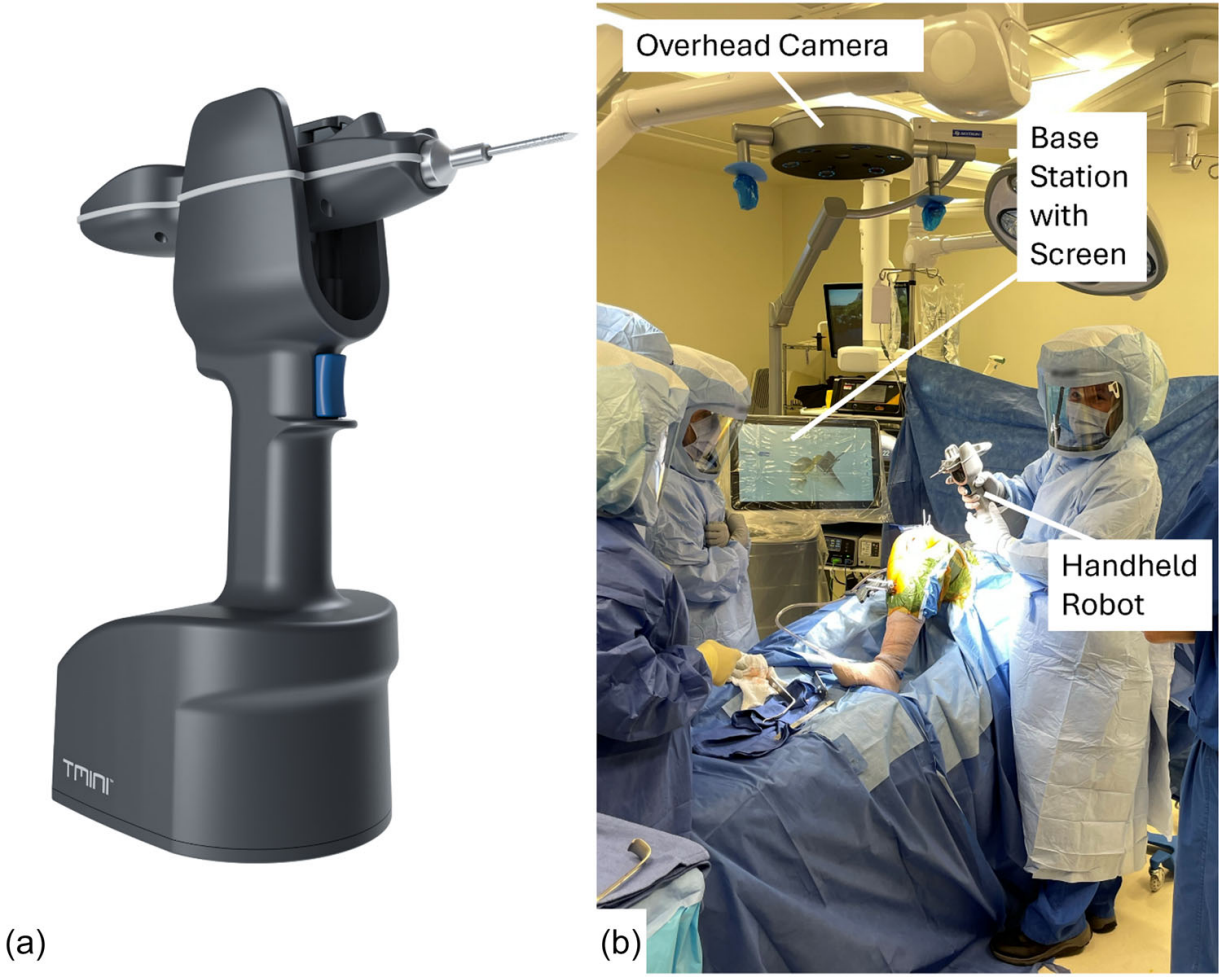
The TMINI® Miniature Robotic system (a new handheld robot). (a) The wireless, handheld robot and (b) shows the handheld robot and the overhead field of view system (camera) being used during a total knee arthroplasty procedure.

The TMINI® Miniature Robotic System (TMINI System, THINK Surgical, Inc.) is a new FDA‐cleared wireless, handheld, open‐implant robot (new handheld robot) that seeks to address the usability challenges of previous rTKA systems (Figure 1) [31]. It utilizes a CT‐based preoperative plan. As a wireless handheld device, it allows for unrestricted robot position relative to the patient, compared to the limited workspace inherent in traditional robotic arm systems. In addition, the handheld robot is autoclavable and, therefore, sterile draping is not required. The TMINI System includes a handheld robot and a base station. The base station utilizes a display and an overhead line‐of‐sight camera that tracks the movement of the handheld robot and allows for traditional bedside patient access. This contrasts with other systems, that use a side‐line‐of‐sight tracking array that may interfere with surgeon and staff positioning by requiring a cleared side of the patient. It is an open‐implant platform, meaning a variety of different implants sold by different companies can be used.

**Figure 2:**
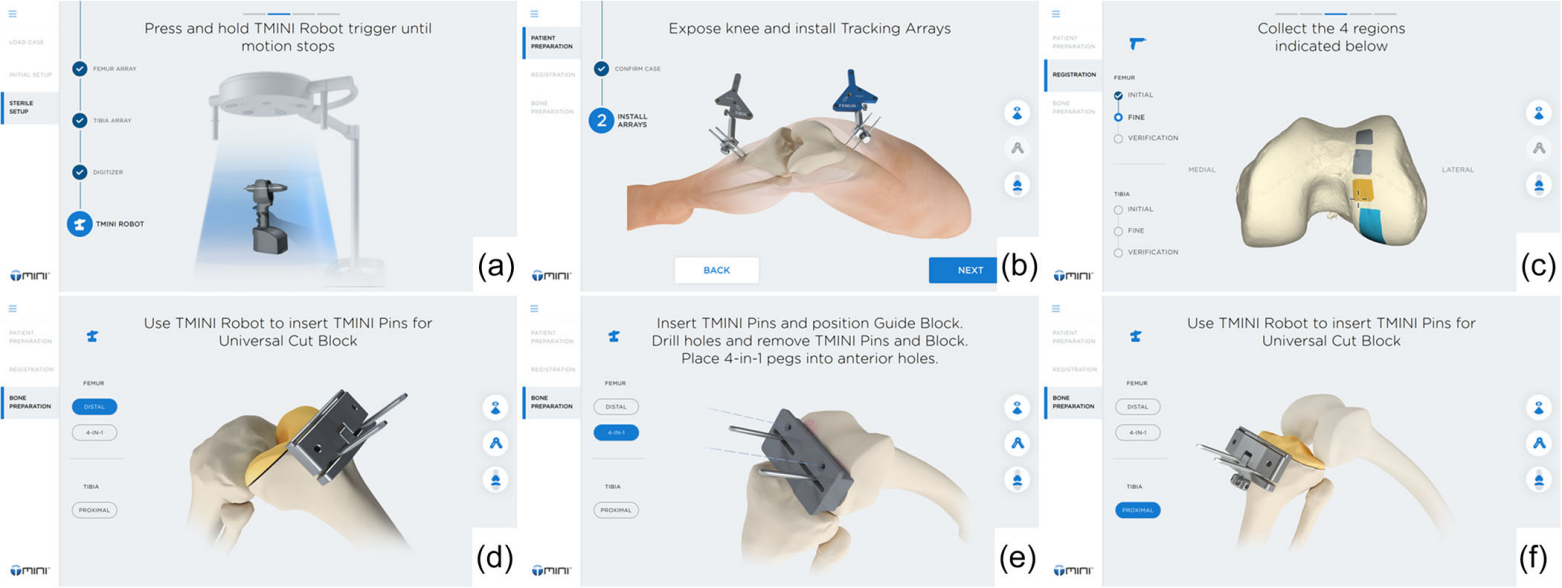
A high‐level overview of the TMINI® Miniature Robotic system workflow. The steps consist of (a) calibration of the tools to the robot, (b) installation of bone tracking arrays in the femur and tibia, (c) registration of the bones to the robotic system and computed tomography‐based surgical plan, and (d–f) placement of pins for cut block placement. Note that femur‐first and tibia‐first workflows are both possible, and the (e) 4‐in‐1 guide block is specific to prepare the bone for the implant manufacturer 4‐in‐1 cut block.

An overview of the surgical workflow as displayed on the base station screen is shown in Figure 2. After the calibration, incision and bone registration, the new handheld robot guides surgeon placement of two bone pins along each of three predefined planes. Prior to placing the bone pins, the TMINI system enables the surgeon to track the ROM and perform balancing by changing the predefined planes, if desired. For two of the three predefined planes, the surgeon secures a cutting guide to the bone pins and completes the distal femoral resection and the proximal tibia resection. For the 3rd predefined plane, a guide block associated with the chosen implant is secured to the bone pins and is used to place the specific implant manufacturer's 4‐in‐1 cut block to enable the remaining femoral resections to be performed. This rTKA procedure follows the traditional manual workflow and bone resections are completed using tools analogous to traditional manual procedures. The preplanned placement of the cutting guides removes the need for the intramedullary or extramedullary rods. The TMINI system allows the surgeon to choose femur‐first or tibia‐first workflows. Intraoperative adjustments and assessments can also be performed during the procedure.

The purpose of the present study was to determine the early outcomes with this novel handheld, wireless robot in patients undergoing TKA. We hypothesize that early outcomes would show no increase in complications and equivalent safety profiles to prior technology.

## METHODS

This study used retrospective patient data with local IRB approval. Clinical results, including the safety and performance of the new handheld robot for the first 25 rTKA cases by a single surgeon using the TMINI system were compared to a concomitant group of 25 mTKA cases performed by the same surgeon (Table 1). All rTKA procedures using the new handheld robot system were performed using the Enovis™ EMPOWR Knee System® (Enovis™), and the surgeon had not used this implant system prior [11]. System setup, time to first bone resection, and skin‐to‐skin times for eight rTKA cases (1, 2, 3, 5, 7, 10, 15 and 25) were recorded and compared to times from two mTKA cases.

**Table 1:** Study cohorts.

Cohorts	rTKA	Manual (mTKA)
Total number of patients	25	25
Average age	74.0	71.5
Gender (#, % male)	11/25 (44%)	9/25 (36%)
Operative side (#, % right)	12/25 (48%)	14/25 (56%)
Average BMI	27.4	28.2
Average ASA	2.26	2.36

Abbrbeviations: ASA, American Society of Anesthesiologists Score; BMI, body mass index; mTKA, manual total knee arthroplasty; rTKA, robotic total knee arthroplasty.

Patients had 2‐, 6‐ and 3‐month follow‐up appointments, with none lost to follow‐up. Patients were monitored out to 3 months for 22 TKA‐related adverse events as outlined by the Knee Society in Healy et al. [13]. Alignment outliers were evaluated by anterior‐posterior (AP, or coronal) weight‐bearing short knee X‐ray and were defined as an HKA angle deviation >3° from the planned alignment [23]. ROM and KSS were performed at the 2‐ and 3‐month visits. Pain at PACU arrival and discharge was also recorded. ROM and KSS between the two groups were compared with a Student's *t*‐test, and Fischer's exact test was used to compare 2 × 2 tests such as gender, operative side and same‐day discharge rate, or patients that arrived home before midnight the day of surgery.

## RESULTS

The results of the study can be seen in Table 2. There were no adverse events in either cohort. Pain in the rTKA cohort was lower than the mTKA cohort at PACU arrival and discharge, and more rTKA patients than mTKA patients were discharged on the day of the procedure. There were no HKA angle outliers in the rTKA cohort and one in the mTKA cohort when the postoperative X‐rays were evaluated by the operating surgeon. There were no significant differences between cohort groups preoperative or postoperative ROM or KSS. There were no manipulations or reoperations in either group. rTKA case 5 was less than 1 min slower than the slowest mTKA case, and rTKA cases 7, 10 and 25 were all equal to or faster than the mTKA cases skin‐to‐skin times. Skin‐to‐first resection times were slower in all rTKA cases, and 6/8 rTKA cases had set‐up times under 8 min.

**Table 2:** Safety and performance outcomes.

Outcomes		rTKA	mTKA	*p* value
Safety	Adverse events	0/25 (0%)	0/25 (0%)	*p* = 1.0
	Skin to skin time (min)	51.1 ± 9.0	46.0 ± 7.1	*p* = 0.031
	Pain (PACU arrival)	0.76 ± 0.9	1.22 ± 1.1	*p* = 0.11
	Pain (PACU discharge)	1.2 ± 1.8	2.1 ± 2.5	*p* = .015
	Same‐day discharge	13/25 (52%)	8/25 (32%)	*p* = 0.25
Performance	HKA outliers (>3°)	0/25 (0%)	1/25 (4%)	*p* = 1.0
	ROM	124.2 ± 6.6	122.4 ± 5.1	*p* = 0.24
	KSS	93.6 ± 5.7	92.2 ± 4.9	*p* = 0.36

*Note*: Eight rTKA cases and two different mTKA cases were measured for skin‐to‐skin time.

Abbreviations: HKA, hip‐knee‐ankle; ROM, range of motion; mTKA, manual total knee arthroplasty; rTKA, robotic total knee arthroplasty.

## DISCUSSION

The intention of this study was to observe the early outcomes of TKA with this novel handheld, wireless robot. Twenty‐five rTKAs and 25 mTKAs were performed. Patient follow‐up was up to 3 months to measure HKA angle and PROMs for performance, and AEs and pain for safety.

Similar skin‐to‐skin times were observed by case 5, with the first faster rTKA case occurring at case 7. Interestingly, the time to initial bone resection was longer in all rTKA cases, indicating that the delay necessary for array placement and registration may be made up later in ease of achieving target bone resections compared to mTKA. The similarity in mTKA and rTKA workflows using this new handheld robot may assist in this. The robotic placement of pins based on preplanned CT images allows cutting guide placement for bone resections, without requiring the intra‐ and extramedullary rods used in manual procedures. Using a handheld robot, followed by standard power tools allows surgeons to use familiar workflows and have usual visibility and tactile feel during bone resection. The overhead camera allows the surgeon and assistants to operate more freely than other rTKA systems, as line‐of‐sight issues are minimized between the camera and robot. This benefit may have helped in achieving OR time neutrality. Interestingly, the rTKA cases were also the initial uses by the surgeon of an unfamiliar implant, yet the robotic procedure still yielded time neutral results. It is possible that there were implant differences that enabled this, but as the surgeon had extensive experience with the mTKA implant and no experience with the rTKA implant, the surgeon would be expected to be much faster and more proficient with the mTKA implant. Staff setup was generally under 8 min. The robotic tool in this case requires no sterile draping, which may have helped it feel more familiar in comparison to draped robotic arms. In many cases, the robot was moved from room to room in flip room settings.

Patient outcomes from the initial rTKA with the new handheld robot showed preliminary outcomes at least comparable to standard mTKA cases. No TKA adverse events were observed in either cohort. It has previously been shown that while mTKA is considered a safe procedure, there are risks, such as periprosthetic fracture and revision, that are elevated in rTKA [13, 32]. None of these, nor others in the standardized list of 22 TKA‐related complications [13], were observed in the patient outcomes from mTKA or rTKA using the new robot.

ROM and KSS had no difference between rTKA and mTKA cohorts. This reflects the existing literature, where no PROM differences are often observed between the two methods [1, 2]. The relatively short timeframe of this study, 3 months, may also be a reason for seeing no difference between cohorts. The lack of difference in ROM is likely due to surgeon skill with either method, though increased ROM after robotic procedures has been shown in other studies [19]. Additionally, it has been shown that differences in implants, such as between the mTKA and rTKA groups, may not have an impact on PROMs [12].

Pain at PACU arrival and discharge were lower in rTKA patients, and the pain increase from arrival to discharge was also smaller in rTKA, but none of these measures were statistically significant. Similarly, more, but not significantly more, patients were discharged on the day of surgery in the rTKA cohort. While not statistically significant, both of these results show a potential improvement in the rTKA cohort from the mTKA, indicating preliminary safe and effective use of the new handheld robot, which is similar to results from prior studies on other rTKA systems [6, 22].

Accuracy has long been touted as one of the main driving factors in the adoption of robotic technology and that is once again held up in this study. There were no coronal HKA alignment outliers in the rTKA cohort with the new handheld robot, and only one in the mTKA cohort. Both of these results are better than the generally reported manual alignment outlier rate of approximately 32%, which is the standard of care for over three quarters of TKA patients [14, 18, 29]. There is a wide range of reported rTKA alignment rates, but many fall within 7%–20% [5, 7, 9, 15, 18, 36]. The alignment rate utilizing the new handheld robotic system presented here, albeit a small sample, puts its accuracy as good or better than other established robotic systems or mTKA.

### Limitations and next steps

This study, while showing preliminary safe and effective patient outcomes, has several limitations. The study was focused on assessing the early adoption and early safety of the technology. The study comprises only the first 25 rTKA cases performed with this system by a single surgeon. This manuscript represents an initial step in the postmarket investigation of this robot. As such, the study is likely underpowered to determine long terms complications and differences in PROMs. It is our hope that larger studies with longer follow‐up are expected as the system is used by independent surgeons. The sample size of 25 is small such that the data presented would benefit from the inclusion of more subjects. A longer follow‐up period may also show a difference between the rTKA and mTKA PROMs. However, longer studies have been found to show that PROMs between rTKA and mTKA procedures merge over time. The safety outcomes of the rTKA cohort related to use of the new handheld robot are unlikely to change at a longer follow‐up period, as the adverse events commonly linked to rTKA specifically, as opposed to other surgical choices, such as longer operative time, infection and periprosthetic fracture, are generally observed during surgery and in the recovery period of generally under 3 months [16, 27].

This study only tangentially focused on the usability or ease of adoption of the system. While the execution of 25 rTKA cases using a new implant is notable, especially considering case times were comparable to manual TKA with a familiar implant at fivecases, this study did not investigate statistically significant measures of the rTKA learning curve, something that has been extensively studied in other systems [4, 7, 24, 30, 34, 35]. It is important to understand how the surgical times change as different surgeons become more experienced. Further research is necessary to understand how surgical times for rTKA using this new handheld robot compare to other robotic systems or to mTKA procedures, adding perspective on the impact of rTKA in the operating room and on clinical outcomes [20, 33]. For example, future studies could consider the use of the same implant in both rTKA and mTKA cohorts to minimize this variable in terms of both surgical times and clinical outcomes. Additional accuracy analysis methods, such as postoperative CT scans, could better assess implant placement. While HKA evaluated by X‐ray is a commonly accepted accuracy measurement [4, 15, 21], short knee X‐ray measurements are less common and a study using CT or full‐length X‐rays to measure other implant degrees of freedom would lead to a more comprehensive assessment of the rTKA clinical accuracy [23, 29]. In future studies, the use of an independent evaluator of the X‐rays would eliminate another potential source of bias.

## CONCLUSION

The present study preliminarily shows safe and efficient early adoption of rTKA with the new handheld robot and an implant new to the surgeon with a limited sample size at a particular institution and follow‐up of 3 months. When comparing the initial robotic cases to experienced manual cases, these limited results yielded similar outcomes. No adverse events or alignment outliers were observed for the rTKA cohort. These preliminary findings suggest that safe integration of the handheld robotic system into clinical practice is feasible; however, larger independent studies are needed to evaluate the device further.

## AUTHOR CONTRIBUTIONS

**Alexander P. Sah**: 30% surgical cases; data collection; writing; review. **James L. Womack**: 10% review. **Yair D. Kissin**: 10% review. **Eytan M. Debbi**: 20% writing; review. **Trevor R. Cotter**: 30% data analysis; writing.

## CONFLICT OF INTEREST STATEMENT

Dr. Alexander Sah is a compensated Medical Director for THINK Surgical, Inc., and Dr. Sah, Dr. Eytan Debbi, Dr. Yair Kissin, and Dr. James Womack are each compensated Surgeon Advisory Board members with THINK Surgical, Inc. Dr. Trevor Cotter is an employee of THINK Surgical, Inc.

## ETHICS STATEMENT

Local IRB, Washington Hospital, Fremont, CA, USA.

## Data Availability

Data are available on request from the authors.
